# Effect of Fermentation on the Nutritional Quality of the Selected Vegetables and Legumes and Their Health Effects

**DOI:** 10.3390/life13030655

**Published:** 2023-02-27

**Authors:** Eliza Knez, Kornelia Kadac-Czapska, Małgorzata Grembecka

**Affiliations:** Department of Bromatology, Medical University of Gdańsk, Gen. J. Hallera Av. 107, 80-416 Gdańsk, Poland

**Keywords:** fermented food, lactic acid bacteria, functional food, fermented legumes, food quality, fermented vegetables

## Abstract

Vegetables are rich in many vitamins, mineral components, antioxidants, and macronutrients. However, they also contain high levels of anti-nutrients, which reduce the bioavailability of some nutritionally essential components such as zinc or iron. The processing of vegetables, including microbial fermentation, reduces or neutralizes tannins and phytates, which results in greater bioavailability of vitamins and minerals. Another effect of bacterial processing is an increased antioxidant potential, which has been linked to the release of polyphenols from complexes with anti-nutritional ingredients. Moreover, there were identified bioactive substances such as γ-aminobutyric acid or inhibitors of an angiotensin-converting enzyme in fermented vegetables. These substances may support the treatment of lifestyle diseases, such as depression or hypertension. However, there are still no data about these substances’ bioavailability after the consumption of fermented vegetables. Additionally, fermented foods are rich in probiotic microorganisms, where the main group is lactic acid bacteria. The effect of fermentation on each vegetable is different. This should be a reason to expand our knowledge as more innovative and untested fermented products appear on the market.

## 1. Introduction

Fermentation has been used by humans for many hundreds of years. In ancient times, Hippocrates noted that “bad digestion is the root of all evil”, and the benefits of fermented foods were known long before Louis Pasteur discovered lactic acid-producing bacteria. The earliest reports of fermented foods date back to 4000–3000 years B.C. and involve mushrooms and soybeans [1]. Throughout history, new fermented products were developed. The newest scientific evidence is emerging of the beneficial effects of fermented products on human health, mainly the gut microbiota. For this reason, knowledge of this process and its products is constantly being expanded.

Fermentation is an inexpensive process requiring little energy to preserve foods, enriching them with newly formed bioactive compounds and improving organoleptic properties, digestibility, and assimilability [2]. If it concerns food, it is acidic fermentation with lactic acid as the main product. It can be divided into spontaneous, backward, and controlled types. The first type occurs spontaneously as a result of the sequential and competitive action of many microorganisms, of which the best adapted strains dominate the microbial environment of the processed product. In a backward type, a portion of the previous batch from successful lactic fermentation is used as a starter culture donor. The controlled process uses specific and previously isolated strains. These usually include different species of lactic acid bacteria, yeasts, and fungi. In all types, different temperatures and salinity percentages of the test sample are used, aiming at optimal values. In the case of sodium chloride (NaCl), it constitutes up to 8–10% of all components. Salt is an inherent substrate of food fermentation, with NaCl mostly used. Recently, there have been tests performed about replacing this compound with potassium chloride (KCl) or monosodium glutamate [3,4]. The high salinity of fermented vegetables may make them unsuitable for everyone, including hypertensive patients [5]. The best conditions of fermentation are established experimentally by finding the most suitable sensory quality, probiotic composition of the product, and enrichment of the substrate with additional health-promoting properties such as increased antioxidant potential [5]. However, there is still little research on this topic [6,7,8,9,10].

The fermentation process can improve the digestibility of nutrients in products of plant origin [11]. It is one of the strategies to reduce the concentration of anti-nutritional compounds, which include salts of phytic acid that are commonly found in plant-based foods [12]. The food processing in question, through the production of the phytase enzyme that degrades phytic acids, improves the bioavailability of the whole product and the mineral components, such as calcium (Ca), zinc (Zn), and iron (Fe) [13]. Additionally, lactic acid bacteria (LAB), in combination with the yeast *Saccharomyces boulardii*, result in the enrichment of fermented products with bioactive compounds such as the isoflavones in soybeans—genistein and daidzein [14]. However, not every fermented product is examined. There are many studies concerning dairy products, and that topic is quite well-known. Still, there are food groups, such as root vegetables, where the influence of fermentation was examined only partially. The best examined of all fermented vegetables are legumes. There is a need to do more research about the impact of fermentation on other groups such as roots or cruciferous vegetables.

**Figure 1:**
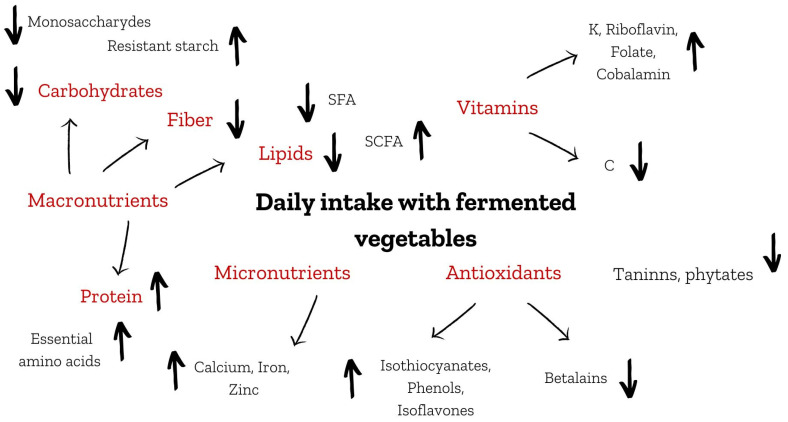
Daily intake with fermented vegetables compared with fresh ones. SFA—saturated fatty acids, SCFA—short chain fatty acids. ↓ Decrease in daily intake of particular nutrient from fermented vegetables compared to fresh ones. ↑ Increase in daily intake of particular nutrient from fermented vegetables compared to fresh ones.

Fermented foods show a variety of positive effects on health, and microbiologically produced new bioactive substances make them functional products [15,16,17,18]. Processing by microorganisms affects the content and bioavailability of many vegetable components such as macronutrients, as well as compounds present in food in small amounts, such as polyphenols and vitamins (Figure 1). The purpose of this article was to summarize current knowledge about fermented vegetables. Moreover, the authors pointed out the knowledge gaps in this topic and marked the need for deepened research. Due to a lack of information, many vegetables were omitted from the article. Legumes, cucumbers, and cabbage were described in the most detail. Other plant products were presented on a smaller scale, which shows the disparity in the research concerning fermented vegetables. In the following part of the article, the influence of microbiological processing of vegetables on the amount of particular substances and the related impact on human health was presented.

## 2. Macronutrients

A comparison of the protein, fat, carbohydrates, and fiber contents in fresh and fermented vegetables was shown in Table 1. Figure 1 presents the changes in daily intake of macronutrients with fermented vegetables compared with fresh ones. Daily intake of the aforementioned nutrients is affected not only by their amount in the fermented sample but also by their bioavailability, which is much higher than in fresh products due to a decrease in anti-nutritional compounds such as tannins or phytates.

### 2.1. Carbohydrates and Dietary Fiber

**Figure 2:**
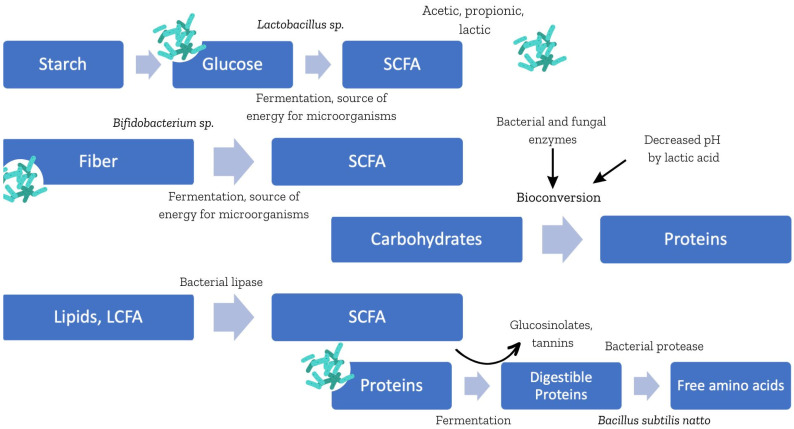
Breakdown of macronutrients into smaller parts during the fermentation process and possible mechanisms. LCFA—long chain fatty acids, SCFA—short chain fatty acids. Grey arrows show the direction of changes and black arrows show factors that influence particular mechanisms.

Plant-based foods are characterized by low levels of all macronutrients, except for legumes. Foods containing high amounts of carbohydrates, such as starch, are causing blood glucose levels to rise. Each specific product has a corresponding glycemic index (GI), which expresses the rate of increase in blood glucose after ingestion. Fermentation reduces GI by converting glucose, which was formed from the breakdown of starch, to short-chain organic acids such as propionic, acetic, and lactic ones. There is also another explanation of this effect that involves a decrease in pH by lactic acid and, consequently, a reduction in the activity of starch hydrolyzing enzymes (Figure 2) [20]. Additionally, the fermentation of carbohydrates-containing vegetables such as legumes results in an increased amount of resistant starch [21]. However, due to the utilization of fiber as a source of energy for microorganisms, the level of this component in the final product is reduced [22]. Fermentation also reduces the amount of monosaccharides by converting them to polysaccharides. Such a relationship was observed in fermented carrot juices, where 52% more polysaccharides and 27% fewer monosaccharides were detected. This was probably due to the enzymatic conversion of simple sugars to insulin by fructosyltransferases produced by starter microorganisms, i.e., *Lactobacillus gasseri* DSM 20604 and DSM 20077 [23].

Conversion of monosaccharides into polysaccharides is an important feature of fermented products that makes them more suitable for diabetic patients. Bacteria from *Lactobacillus* species seems to be the most efficient in this process. Moreover, because of their probiotic potential, short-chain organic acids are important substances for human health, especially for gut microbiota [24]. There is a need to analyze their bioavailability to microbiota and the production of short-chain fatty acids (SCFA) after the consumption of the fermented product. However, most vegetables (except legumes) are characterized by a small amount of carbohydrates per 100 g of product [25]. This is probably why the majority of the research on this topic focuses on foods such as soy or fava beans. Increased demand for these products by consumers and the possibility of synthesizing new bioactive compounds from mono and disaccharides are the main reasons for expanding that knowledge [23].

### 2.2. Proteins

The amino acid profile is less optimal in plant products than in animal ones. This is due to the lower content of lysine and sulfur-containing amino acids such as methionine and cysteine. However, with a well-balanced vegan diet, the requirement for all amino acids is fulfilled [26]. In addition to the individual amino acid composition, the protein quality is also determined by the percentage of its digestibility. Legumes are the primary source of proteins in a vegan diet [26,27]. Purified and concentrated compounds, such as soy proteins and gluten, are characterized by a relatively high digestibility. However, anti-nutritional compounds, tannins, glucosinolates, or isothiocyanates that reduce the bioavailability of the macronutrients are also present in fresh products [26]. During controlled fermentation with *Lactiplantibacillus plantarum* C48 and *Levilactobacillus brevis* AM7 strains, the free amino acid (FAA) content increased in all legumes studied, which was connected with a decrease in tannins [28]. Due to the high amount of proteins in soybeans, a significant increase in FAA percentage (from 3.74 to 64.30 μg/mL) was observed. This was likely related to the active protease produced by *Bacillus subtilis* natto (Figure 2) [16]. In another study that examined fermented lentils, it was noted that the amount of digested protein was higher [21]. A study comparing yam bean flour before and after the fermentation process showed an increase in protein from 21.33% to 25.02% on a dry basis. Moreover, some essential amino acids, such as leucine, increased by 0.16%, and isoleucine increased by 0.31%. However, decreased content of valine (0.87% less) was observed [29]. Another example is fermented chickpea flour, which features a more available lysine than the one in the original product [30]. However, legumes are not the only products in which an increase in protein bioavailability has been noted. Such an effect was also observed in fermented bamboo shoots [31] and cucumbers [32]. The greatest increase was observed for the amino acids such as lysine [(from 28.7 to 110.9 mg/kg fresh weight (f.w.)], isoleucine (from 38.9 to 162.2 mg/kg f.w.), and leucine (from 38.9 to 129.8 mg/kg f.w.) [32].

Fermenting legumes with *Bacillus subtilis* natto may improve plant protein digestibility and the amount of essential amino acids, which may be important for vegans. Moreover, there should be more studies about the influence of fermentation on this aspect, and scientists have to find the best conditions to obtain the best quality protein. Some proteins, such as Api g in celery or Gly m 1 and Gly m 2 in soy beans, are allergens [33]. Protein denaturation to FAA can be a way to decrease allergenicity, but that knowledge should be expanded [34,35]. Future challenges need to concentrate on analyzing vegetables being allergens, such as celery. Fermentation may be helpful in nutrition diversification by people with various allergies, including cross ones. It could prevent many deficiencies that people on elimination diets are exposed to [36].

### 2.3. Lipids

Besides proteins, fermentation also affects fat content by reducing its amount. Some attribute this fact to the metabolism of microorganisms in the processed product, the breakdown of lipids by lipase, and their use as an energy source for bacteria [37]. Additionally, a higher amount of SCFA has been reported in fermented soybeans than in the unfermented sample. The main SCFA in the final product was acetic acid. Its concentration increased from 2.90 to 39.89 μmol/g during 0–72 h fermentation. However, SCFAs such as butyric and valeric acids were not detected in all the samples [16]. 

There is a lack of research regarding the impact of fermentation on lipids located in a fresh sample. Except for soybeans, vegetables are not products with high content of this macronutrient. However, knowledge concerning other silages should be expanded, and research should perform processes regarding all macronutrients. 

## 3. Micronutrients vs. Anti-Nutritional Compounds

Fermentation can also affect mineral content (Table 2). Although legumes constitute a rich source of vitamins and minerals, these compounds are characterized by low bioavailability. This is due to the presence of many anti-nutritional compounds such as phytates and myo-inositol. Fermentation can reduce the amount of these substances, thus contributing to the increased bioavailability of minerals [38]. Food products such as fermented chickpea flour are characterized by less phytic acid and tannins, which may also improve the bioavailability of minerals [30]. There was a significant difference observed in the elements’ content in fermented mung bean compared to the product before processing [37]. Calcium and Fe were determined, in increased concentration, from 21.5 and 43.45 mg/100 g to 49.30 and 56.50 mg/100 g, respectively. However, Zn content decreased from 3.80 to 3.05 mg/100 g. Additionally, the bioavailability of Fe and Zn, tested in vitro, was significantly (*p* < 0.05) higher in products fermented with *Aspergillus awamori* strain. That was due to decreased content of phytates (from 0.4 to 0.11 mg/100 g) and saponins (from 0.18 to 0.03 mg/100 g) [37]. The discussed processing using *Aspergillus oryzae* MTCC 3107 also increased the assimilation of Fe and Zn, which almost doubled, after 96 h fermentation, from 17.25% to 30.22% and from 14.36% to 29.63%, respectively [39]. A similar observation was done in the case of Fe and Zn contents in fermented black pea flour after 96 h of microbiological processing. The amount of these elements increased from 32.892 to 38.421 µg/kg and from 18.328 to 24.901 μg/kg, respectively [39]. The processing discussed here may increase the bioavailability of microelements despite their lower content [40]. During fermentation, microorganisms produced specific enzymes that helped in the hydrolysis of insoluble complexes of elements with phytates and polyphenols. However, lower amounts of elements such as Fe, phosphorus (P), magnesium (Mg), and Ca in fermented bamboo shoots were noted [31].

It is difficult to find similarities in micronutrient changes. They are mostly determined by the fresh sample used. However, several patterns can be recognized. Primarily, fermented vegetables are characterized by higher bioavailability of elements compared to products before processing. That is due to a decrease in anti-nutritional compounds such as tannins and phytates. The absolute quantity of particular microelements in fermented vegetables is characterized by various changes (increases or decreases). However, their bioavailability is always higher, resulting in a greater daily intake of essential elements via fermented products compared to fresh vegetables. 

There is plenty of research analyzing the content of microelements in fermented products and their bioavailability [41,42]. However, there are a few studies performed concerning some groups of vegetables, such as root vegetables. There is an urgent need to analyze the impact of fermentation on the content of the micronutrients and their bioavailability in the group of fermented root vegetables. As of today’s knowledge, it is not possible to indicate the best conditions of fermentation that allow for obtaining the highest bioavailability and quantity of macronutrients. Undoubtedly, there should be still research performed concerning the process in question in order to gain this information.

## 4. Vitamins

As with minerals, in the case of vitamins, there are conflicting results related to the increase or decrease in their content in the final product.

Vegetables are characterized by high vitamin content, depending on the type of raw material. For instance, *Cruciferous* vegetables are rich in vitamin K, while *Solanaceous* vegetables, such as peppers, contain significant amounts of vitamin C [43]. Fermented vegetables can contain a high quantity of vitamin K, which can be produced by some probiotic strains such as *Bacillus subtilis* natto [44]. The process of food decomposition by microorganisms may additionally cause changes (positive and negative) in the content of other vitamins. That may depend on the specificity of the raw material. 

Fermented legumes, such as cowpea and bean, were characterized by a decrease in thiamine but an increase in riboflavin content [45,46,47]. Another study reported not only an increase in riboflavin concentration but also in α-tocopherol and thiamine in fermented kidney beans and peas [44,48]. Fermentation can also increase folic acid and cobalamin levels in soybean products [49]. These changes are likely related to the metabolic activity of starter cultures, such as *Bacillus subtilis*, used for natto production [50]. A variety of changes were noted in the commonly used leafy vegetables in Nigeria—*Amarathus hybridus* (Bush green), *Telfairia occidentalis* (Fluted pumpkin leaf), *Vernonia amygdalina* (Bitter leaf), and *Pterocarpus mildbraedii* (White camwood leaf) [51]. There was an increase in both niacin (1.32–1.62 mg/mL) and thiamine (1.37–1.52 mg/mL) contents observed in *Telfairia occidentalis* due to the fermentation process [51]. However, a decrease in vitamin C was noted in all plants. *Pterocarpus mildbreadii* was an exception, as there was a slight increase recorded in the content of ascorbic acid (0.08–0.13 mg/mL). Almost all vegetables studied showed a time-dependent decrease in vitamin C content during fermentation [51]. Moreover, the same effect was observed for carotenoids, thiamine, riboflavin, niacin, and α-tocopherols in fermented common beans, lupins, and mung beans [37,52].

Sauerkraut is valued for its health-promoting properties [53]. It has a strong anti-inflammatory effect, owing to its high vitamin C and E content [54]. However, less ascorbic acid was determined in the product after processing by microorganisms than in the fresh one. The vitamin C content depends on the brine used and the specific strain. The lowest concentration of ascorbic acid (156.27 mg/100 g) was determined in the product subjected to natural fermentation with a lower NaCl content (0.5%). The highest vitamin C level (266.25 mg/100 g) was observed after processing by *Lactiplantibacillus plantarum* using 1.5% NaCl [55].

In the case of vitamins, results are divergent. When the amount of B vitamins increased, the amount of vitamin C decreased. Moreover, content of vitamin C was associated with NaCl concentration. Interestingly, the highest amount of this bioactive substance was noted in the average concentration of NaCl, while the minimal quantity was identified in the lowest concentration. A decrease in the content of vitamin C after the fermentation process is one of the unfavorable features of this process. However, it turns out that, by manipulating the conditions, this loss can be reduced. Other vitamins, such as K, thiamine, and cobalamin, are characterized by higher quantities in fermented vegetables compared to fresh samples. That is due to the microorganisms present in silages that are able to produce vitamins during fermentation. Until today, an adequate mechanism for this process had not been discovered. Only a few groups of fermented vegetables have been studied in terms of vitamin content. There is a lack of results concerning the fermentation of root vegetables, tomatoes, and vegetables from the *Brassicaceae* family. Future studies should be focused on these products.

## 5. Antioxidant Potential

### 5.1. Antioxidants Content

The antioxidant potential of fermented vegetables depends on the compounds from the group of phenols, flavanols, polyphenols (e.g., ascorbigen, indole-3-carbinol) and vitamin C. The effect of fermentation on the composition and presence of individual substances can vary considerably depending on the specificity of the product and the starter culture used.

Sauerkraut is probably the most popular fermented vegetable [19]. Important components of sauerkraut, in terms of its antioxidant potential, are isothiocyanates, ascorbates (ascorbigen), and indole-3-carabinol formed from the breakdown of glucosinolates [54,56]. These compounds show antioxidant, anticancer, and anti-inflammatory properties [57,58]. Their amounts increase during processing by microorganisms [55]. The content of isothiocyanates is affected by fermentation time and storage period. The highest levels of all studied compounds in sauerkraut were observed on the 4th–5th day of the process, while after this period, their amount decreased time-proportionally. The concentrations of ascorbigen, indole-3-acetonitrile, and 3,3-diindole were higher even at the end of the experiment compared to the initial value. The exception was indole-3-carbinol for which content decreased [59]. In sauerkraut juice, the concentrations of these substances also increased in the first days but then decreased slightly with time [56]. Sauerkraut enriched with selenium (Se) can be considered a functional food. This element causes additional growth of LAB and a significant increase in indole-3-carbinol and indole-3-acetonitrile. Extracts from selenium-enriched sauerkraut showed higher antioxidant activity than extracts from the unenriched product and inhibited nitric oxide production in lipopolysaccharide-induced macrophages [40,59,60].

The amount of flavonoids and total phenolic content (TPC) in fermented bamboo shoots increased from 49.69 to 59.43 mg catechin equivalent/100 g (CE/100 g) and 29.0 to 42.0 mg gallic acid equivalent/100 g (GAE/100 g) f.w., respectively [31]. This increase may be due to the hydrolysis of glycosidic bonds in phenolic compounds by enzymes synthesized by fermenting microorganisms, leading to the release or formation of various bioactive compounds, i.e., protocatechuic acid, p-hydroxybenzoic acid, and syringic acid [31]. Usage of *Lactococcus lactis* strain resulted in 113% increase in phenols and 147% in flavonoids. The increase in TPC in the fermented product is probably related to the synthesis of enzymes such as β-glucosidase, which can catalyze the release of phenols. Such a case was reported during the analysis of soy beverage [61]. However, LAB strains and lactic acid do not always increase the content of phenols. There are pickled vegetables, such as fermented tomatoes, which are characterized by lower TPC than in fresh samples [62]. Total phenolic content measured by the Folin–Ciocalteu method amounted to 2.4 mg GAE/mL for extracts from fresh tomato and 1.6 mg GAE/mL for fermented tomato [62].

Legumes are characterized by a high content of substances showing antioxidant potential. During the fermentation of lentils, an increase in the level of polyphenols from 2.1 to 3.2 mg GAE/g dry weight (d.w.) was observed [21]. The total content of phenolic compounds can increase by over 100%. The use of *Aspergillus awamori* as a starter culture allowed the formation of new phenolic compounds such as cinnamic acid [63]. Fermented legumes were characterized by an increased general phenolic content and antioxidant activity [28].

The opposite changes, i.e., a decrease in the content of biologically active components, were observed in pickled beets. In this product, the highest antioxidant activity is mostly expressed by pigments from the betalain group. Fermentation caused a decrease in betalains content by 61–88%, which resulted in a lower antioxidant capacity. This decrease was limited when the vegetables were not previously peeled [64].

In general, fermentation causes an increase in TPC and total flavonoid content (TFC) levels. This is due to lactic acid bacteria used in controlled fermentation or evolving in a spontaneous one. The production of the enzyme β-glucosidase by them is responsible for a decreased quantity of anti-nutrients located in complexes with antioxidant compounds. However, antioxidant content may also decrease (e.g., betalains in pickled beetroot). Unfortunately, there is a lack of standardized methods used to determine antioxidant potential [65]. That is why it is difficult to compare the antioxidant effects of particular products. This is an area that requires a great deal of solid research to determine the best fermentation conditions for all vegetables.

### 5.2. Antioxidant Potential

The majority of vegetables are characterized by an increase in antioxidant potential after the fermentation process, which is mainly related to a decrease in the amount of anti-nutritional compounds and the release of antioxidants from the complexes [12]. However, as in the case of vitamins and minerals, these changes may vary depending on the raw material’s specificity. Both the antioxidants content and potential of fermented vegetables depend, mostly, on raw material (Table 3). However, there are only a few studies concerning other vegetables, such as root vegetables or plants, from the *Brassicaceae* family [66,67,68]. *Brassicaceae* constitutes a source of glucosinolates (GLS), which are anti-nutritional compounds, but during fermentation, their content decreases. Then, breakdown products of GLS are formed. Those are compounds responsible for antioxidant potential (e.g., indol-3-acetonitrile) [69].

The antioxidant potential also changes after fermentation. Hydroxyl free radical scavenging activity (DPPH) and total antioxidant capacity were higher, while tannin content was lower, in fermented mung bean [37]. Fermented kidney beans showed a 39.5% increase in antioxidative activity associated with three times higher TPC [37]. The free radical scavenging potential, assessed by ferric-reducing antioxidant power (FRAP), DPPH, and Trolox equivalent antioxidant capacity assay (TEAC), was also higher in fermented bamboo shoots, i.e., 2.13, 5.76, and 6.24 µmol Trolox equivalent/g (TE/g) compared to 1.72, 5.71, and 5.82 µmol TE/g in fresh shoots, respectively. The increase in free radical scavenging activity during fermentation was proportional to the TPC, confirming that they contribute to the antioxidant activity [31].

Undoubtedly, a common feature of fermentation is an increase in antioxidant potential due to a decrease in anti-nutritional compounds and the release of phenols and other antioxidants from complexes with these components. However, the specificity of changes varies and is dependent on a variety of factors—most notably, the type of fresh sample used (vegetable). It is important to gain knowledge of how antioxidants’ content, form, and bioavailability change during the process in question [70]. Moreover, the methodology of antioxidant potential analysis is not standardized [65]. Therefore, the comparison of the results from several studies may be difficult due to the different materials and methodologies used by researchers. There is an urgent need for the development of a standardized method for evaluating antioxidant potential.

**Table 3:** Effect of the fermentation process on the content of mineral components, anti-nutritional and bioactive compounds, and their bioavailability in the select vegetables.

	Content of					
Product	Mineral Components	Anti-Nutritional Compounds	Vitamins	Bioactive Compounds	Bioavailability	Source
Fermented bamboo shoots	↓ Ca, Mg, Mn, K, Zn, Fe	↓ phytic acid	n.d.	↑ phenols, flavonoids	↑ Fe	[31]
Sauerkraut	↓ Ca, Mg, Zn, Fe	↓ GLS	vit. C ↓	↑ indol-3-acetonitrile, indol-3-carbinol, ascorbigen ↑ TPC	↑ indol-3-acetonitrile, indol-3-carbinol	[19,54,55,56,71]
Cucumber, pickled	↑ Ca ↓ Mg, Zn, Fe	n.d.	↑ riboflavin ↑ folate, total	↑ ACE inhibitors ↑ GABA	↑ FAA (lysine, leucine, isoleucine)	[19,57,70,72]
Legumes, fermented, other than soya	↑ Fe, Ca ↓ Zn	↓ tannins ↓ phytic acid	↑ riboflavin ↓ thiamine	↑ phenols, polyphenols	↑ Fe, Zn ↑ FAA (lysine)	[29,37,45,73]
Soya, fermented	n.d.	↓ phytic acid	↑ riboflavin ↑ cobalamin ↑ folate, vit. K	↑ phenols ↑ GABA ↑ genistein ↑ daidzein ↑ aglicons ↑ SCFA	↑ isoflavones ↑ phytoestrogens ↑ Zn ↑ protein, FAA	[16,47,49,74,75]

ACE—angiotensin-converting-enzyme; FAA—free amino acid; GABA—γ-aminobutyric acid; n.d.—no data. ↑ Increase in content/bioavailability of particular nutrients from fermented vegetables compared to fresh ones. ↓ Decrease in content/bioavailability of particular nutrients from fermented vegetables compared to fresh ones.

## 6. Other Bioactive Compounds

### 6.1. Inhibitors of Angiotensin-Converting Enzyme

Fermenting microorganisms can contribute to the formation of many bioactive compounds (Table 3) such as peptides that inhibit angiotensin-converting enzyme (ACE). Increased amounts of peptides, such as isoleucine-proline-proline (0.42–0.49 mg/kg), leucine-proline-proline (0.30–0.33 mg/kg), and valine-proline-proline (0.32–0.35 mg/kg), were observed in both spontaneous and controlled fermented cucumbers. In addition, the content of the ACE inhibitor lysine-proline increased three to five times in pickled cucumbers [70]. Fermented red beans also showed ACE inhibitory activity, with an IC50 value of 0.63 mg protein/mL [72].

ACE inhibitors show high therapeutic potential in hypertension, atherosclerosis, and heart failure [76,77,78]. It is important to gain knowledge about the contribution of these compounds in the daily diet and their link to prevention of diseases, as mentioned. There have been few studies on the amount of ACE inhibitors in fermented vegetables [71,73,79]. This is an open area for future research.

### 6.2. GABA—γ-Aminobutyric Acid

Γ-aminobutyric acid (GABA) is another bioactive compound found in pickled vegetables [79]. This component can also be determined in fresh products (in cucumbers at 0.83 mmol/L), but after the fermentation process, its concentration increased (1.21 mmol/L) and remained stable during 6 month storage. The highest level of GABA, i.e., 1.32 mmol/L, was determined in products for which low salt concentration (2% NaCl) was used in fermentation and products prepared for direct consumption [57]. An increased amount of GABA compared to the fresh product was also determined in fermented soybeans [47]. 

Another product rich in GABA is kimchi. During the fermentation of this product, the concentration of GABA increased inversely proportional to the glucose and fructose content. These free sugars are the primary energy sources for GABA-producing bacteria. The highest concentration of the described compound was reached after 20 days of fermentation [79]. The species *Lactobacillus buchneri* was considered the most important producer. The maximum production of GABA in kimchi inoculated with *L*. *buchneri* was 5.83 mg/mL at pH 4.2. The amount of this amino acid was 8 times higher in kimchi produced by controlled fermentation than by spontaneous fermentation and reached 61.65 mg/100 g of product. Other LAB strains producing GABA are *Lactobacillus* sp. OPK 2-59, *Lactiplantibacillus plantarum* kctc103, *Levilactobacillus brevis* kctc 41028, *Levilactobacillus brevis* kctc41029, and *Lactobacillus zymae* GU240 [47,79,80,81].

Г-aminobutyric acid is an important neurotransmitter. Its higher amount in fermented vegetables can be used to enrich the diet of people with a decreased synthesis of this compound, i.e., with depression [82,83]. Lactic acid bacteria, especially *Lactobacillus buchneri* species, are considered the best producers of GABA. There is a need to expand the knowledge of which fermented vegetables are characterized by the greatest concentration of GABA and which types of microorganisms are the best producers of this compound. Moreover, more data is needed to establish where and how this substance is acting after the consumption of GABA-enriched fermented vegetables.

### 6.3. Isoflavones and Phytoestrogens

Isoflavones and phytoestrogens are the characteristic features of legumes, especially soybeans and their products [84]. Due to the presence of conjugated bonds with sugars, their bioavailability is limited. Synthesis of the β-glucosidase enzyme and hydrolysis of conjugated bonds in 60–70% of all isoflavones increases the bioavailability of these compounds. This is possible due to the activity of some bacterial species such as *Lactobacillus acidophilus*, *Bifidobacterium lactis*, or *Lacticaseibacillus casei* [85].

Similar to antioxidants, the amount of bioavailable isoflavones and phytoestrogens increased while the quantity of anti-nutritional compounds decreased. This is a characteristic property of food products after the fermentation process [85]. However, there are substances characteristic of legumes, especially soybeans [84]. Due to their estrogenic activity, they are thought to have anti-cancer properties [14]. There is a need to deepen our knowledge about the best conditions for fermentation, where the amount and bioavailability of these compounds would be the highest.

## 7. Microbiological Composition of Fermented Vegetables

Fermented products of plant origin are characterized by microbial richness, but the particular composition depends on the original raw material (Table 4). In addition, the presence of microorganisms in pickled vegetables is affected by the type of fermentation (spontaneous or controlled) and factors such as the level of salinity or the temperature.

### 7.1. Fermented Roots

Many *lactobacilli* have been identified in root vegetables such as beets and carrots. The analysis 16S rRNA PCR revealed the presence of species such as *Lactiplantibacillus plantarum* and *Lactiplantibacillus pentosus* [86]. Other LAB strains isolated from fermented beets were *Lacticaseibacillus rhamnosus* cek-R1, *Lacticaseibacillus paracasei* subsp. *paracasei cek-R2*, and *Lentilactobacillus otakiensis* cek-R3 [87]. In addition, fermented root vegetables, due to lactic acid-producing bacteria, showed increased inhibition of pathogenic strains such as *Listeria monocytogenes* and *Staphylococcus aureus* [88].

Fermented root vegetables are probably the least analyzed group of products. This is a mistake because they are becoming more and more popular in the consumer’s world. This is a reason why future research about food fermentation should focus on root vegetables.

### 7.2. Sauerkraut

As a result of fermentation, sauerkraut is obtained, which is characterized by many different properties from the fresh product. The most commonly used microorganism species used in its production are *Lactobacillus* spp., *Leuconostoc* spp., and *Pediococcus* spp. Lactic acid bacteria enable a low pH of the final product, which makes it more resistant to pathogenic bacteria [89]. Sauerkraut contains numerous microorganisms such as *Lacticaseibacillus casei*, *Lactobacillus delbrueckii*, *Staphylococcus epidermidis*, *Lactobacillus curvatus*, *Lactiplantibacillus plantarum*, *Levilactobacillus brevis*, *Weissella confusa*, and *Lactococcus lactis* [90,91,92]. Spontaneously fermented cabbage also differs in the microbial composition, as *Firmicutes* type was shown to increase the most, while *Bacteroidetes*, *Actinobacteria*, and *Cyanobacteria* decreased [93]. Another important strain commonly found in sauerkraut is *Lacticaseibacillus paracasei* HD1.7, which produces a broad-spectrum bacteriocin [89]. Moreover, during analyses of the microbial composition of Chinese sauerkraut, *Enterobacter* and *Pseudomonas* bacteria were found to be less abundant in the final product [94]. *Listeria monocytogenes* and *Escherichia coli 0157:H7* were identified in fresh samples of cabbage. However, at the end of fermentation, neither pathogen had detectable populations [95].

### 7.3. Kimchi

Kimchi, a popular Asian fermented vegetable, is rich in *lactobacilli* through the participation of LAB in processing [40]. The most abundant genera include *Leuconostoc*, *Weisella*, *Lactobacillus*, and *Pediococcus* [96]. The initial abundance of LAB in kimchi increased with fermentation time, with the maximum concentration on the 19th day of the process, when it reached about 1.4 × 10^9^ colony-forming unit per ml (CFU/mL). Interestingly, after 19 days of fermentation, the abundance of yeasts belonging to *Saccharomyces* increased in parallel with the decrease in bacterial abundance and reached a maximum value of about 1 × 10^7^ CFU/mL on the 45th day of fermentation [79]. In addition, LAB, by producing lactic acid and lowering the pH of the product, kills *Escherichia coli*. As a result, the content of these pathogenic microorganisms can be below the detection threshold [97].

### 7.4. Pickled Cucumbers

Pickled cucumbers are also rich in LAB. Lactic acid bacteria and the species *Lactiplantibacillus plantarum*, *Lactiplantibacillus pentosus*, and *Levilactobacillus brevis* are the dominant genus in this product. Such abundant LAB has been linked to the bloating effect causing voids in pickles [98]. This effect can be associated with the presence of bacteria of the genus *Leuconostoc*, with the most frequently identified strains—*L. lactis* 1.2.28, *L. holzapfelii* 3.8.12, *L. Fallax* 1.2.22, and *L. mesenteroides* 1.2.47 [99]. However, the microbial composition may change over time. With prolonged fermentation time, the lactic acid produced by LAB is degraded by other opportunistic microorganisms such as *Clostridium bifermentas* and *Enterobacter cloacae*. During this process, short-chain fatty acids, such as acetic, propionic, and butyric acids, are formed [100].

### 7.5. Fermented Soy Products

Starter microorganisms can also determine the formation of a particular food product. Soybean is a vegetable that yields different end products depending on the fermentation process. Microbial decomposition of cooked soybeans by *Bacillus subtilis* results in natto, while using mixed starters of the genus *Rhizopus* results in tempeh [101]. Pathogenic bacteria such as *Klebsiella pneumonia*, which is a common cause of pneumonia and hospital infections, can also multiply during soybean fermentation. Its presence has been detected in soy sauce, but the health significance of this fact requires further study [94]. However, *Stenotrophomonas*, the typical pathogen found in the control, was reduced and replaced by *Bacillus* during fermentation [102].

Fermented soy products, sauerkraut, kimchi, and pickled cucumbers are probably the best-analyzed of all silages. There are only a few studies discussing the microbial composition of other vegetables after a fermentation process, such as roots or cruciferous vegetables [103,104,105]. That knowledge should be expanded by performing microbial analyses of every fermented vegetable species.

**Table 4:** Microbiological profiles of the selected fermented products.

Vegetable	Microorganisms	Source
Sauerkraut	*Lactiplantibacillus plantarum*; *Lactobacillus*: *delbrueckii*, *curvatus*; *Staphylococcus epidermidis*; *Levilactobacillus brevis*, *Lacticaseibacillus*: *casei*, *paracasei HD 1.7*; *Weissella confuse*; *Lactococcus lactis* *Leuconostoc*: *citreum*, *mesenteroides*	[71,91,92,93]
Beetroot, pickled	*Lactiplantibacillus*: *plantarum*, *pentosus*; *Lacticaseibacillus*: *rhamnosus*, *paracaseil*; *Lentilactobacillus otakiensis*	[86,87]
Kimchi	*Leuconostoc*: *mesenteroides lactis*, *gasicomitatum*, *gelidum*, *karnosum*, *inhae*, *kimchii*, *miyukkimchii*; *Lactobacillus*: *kimchii*, *kimchiensis*, *koreensis*; *Levilactobacillus brevis*; *Lactococcus kimchi*; *Weissella koreensis*	[96,106]
Cucumber, pickled	*Lactiplantibacillus*: *plantarum*, *pentosus*; *Levilactobacillus brevis*; *Leuconostoc*: *lactis*, *holzapfelii*, *fallax*, *mesenteroides*	[97,98,99,100]
Soy sauce	*Bacillus* spp., *Weissella* spp., *Klebsiella*: *variicola*, *pneumonia*	[92]
Carrot Kanji, pickled	*Lactiplantibacillus*: *paraplantarum*, *pentosus*	[86]
Carrot, pickled	*Enterobacteriaceae*: *Erwinia*, *Serratia*; *Leuconostac* spp.; *Lactococcus* spp.	[107]
Radish, pickled	*Enterobacteriaceae*: *Erwinia*, *Serratia*; *Leuconostoc* spp.; *Lactococcus* spp.	[107]
Pepper, pickled	*Enterobacteriaceae*; *Lactococcus* spp.; *Bacillus* spp.; *Leuconostoc* spp.; *Levilactobacillus brevis*	[107]
Tempeh	*Rhizopus*: *oligosporus*, *oryaze*; *Enterococcus faecium*; *Leuconostoc*: *lactis*; *Lactobacillus delbruecki* spp. *Delbrueckii*; *Alicyclobacillus* spp.	[108,109]

## 8. Health Effects of Fermented Vegetables

### 8.1. Probiotic Function

Fermented foods are considered functional probiotic foods, but in vivo studies often show conflicting data. Additionally, such studies are mostly characterized by very small study group sizes. This can be important for the generation of a statistical error [110,111].

In an experiment where scientists compared the effects of 50 g natto daily for two weeks in a group of eight healthy volunteers, a higher content of *Bifidobacterium* and *Bacilli* in the stool was noted [111]. The effect of 60 g of kimchi daily, for 4 weeks among 6 subjects with *Helicobacter pylori* infection, was described. An increased amount of *Lactobacillus* and *Leuconostoc* bacteria in the feces was found [110]. In addition, a lower amount of *Enterobacteriaceae* in the same biological material was observed [112].

Furthermore, oral administration of sauerkraut juices to rats induced probiotic effects where the microorganisms showed high tolerance to low pH. In addition, an antimicrobial effect of this product was reported due to the presence of bacteriocin paracin 1.7 [113]. Prevention of Caco-2 cell adhesion and invasion by *Salmonella enteritidis* was also demonstrated for sauerkraut. This effect was associated with the induction of TNF-a and IL-12 expression by *Lactiplantibacillus plantarum* P2. However, a randomized double-blind controlled trial reported no change in gut microbiota composition after the consumption of 75 g of sauerkraut per day for 6 weeks. However, this experiment was conducted in a group of 58 people with irritable bowel syndrome, where lower scores were reported in patients on the irritable bowel syndrome-symptom severity scale (IBS-SSS) [91].

The survival rate of microorganisms in the gastrointestinal tract from probiotics in special capsules resistant to hydrochloric acid probably never achieves 100% [114,115]. All kinds of foods containing probiotic strains are not characterized by high survival rates of bacteria through the esophagus, stomach, and intestines. However, several studies on the viability of probiotic strains in food have been conducted. A few factors, both food-related (storage conditions, pH, type of packaging) and physiological (pH or bile acids) in the digestion process, influence the nutritional value and microorganism diversity of the final product. Moreover, it was confirmed that bacteria typically lose their viability at temperatures higher than 65 °C [114,116]. Researchers tried to find a way to improve the survival of probiotics in food, but these studies aren’t connected with vegetables [117,118]. This is a broad and open field of research that requires enhanced knowledge. Despite that, consumption of fermented foods, including vegetables, exhibits probiotic potential and confers health benefits on the host [53,119].

### 8.2. Immune Function

The microbial composition of foods may be more important than just for the gut microbiome. Lactic acid bacteria found in abundance in fermented foods may be a preventive factor against cancer [120]. Consumption of fermented products has been shown to have beneficial effects on the immune system. Immunomodulatory effects can even be modeled at the gene level [42,121,122].

Soybean fermented with *Lactobacillus helveticus* R0052 and *Streptococcus thermophilus* R0083 decreased the expression level from 40 to 33 pro-inflammatory genes regulated by TNF-α. Consumption of the fermented product attenuated the expression of genes encoding several pro-inflammatory cytokines, including interleukin 8 [123]. Different *Lactobacillus* species can also regulate the immune response at the cellular level, where they affect the modulation of the Th1/Th2 balance of T helper (Th) cells. Strains isolated from kimchi, such as *Lactiplantibacillus plantarum* CJLP55, CJLP56, CJLP133, and CJLP136, induced the production of more Th-cytokine and interferon-γ (IFN-γ) but less Th2 cytokine and interleukin-4 (IL-4) by macrophages stimulated with these bacteria. Such results suggest that *Lactobacillus* species from kimchi can modulate the Th1/Th2 balance by activating macrophages in a hypersensitivity response induced by Th2 cells [124]. Other *Lactobacillus* and *Bifidobacterium* species from kimchi increase natural killer cells’ (NK) activity, stimulate interferon-β production, and increase secretory immunoglobulin A (sIgA) production [106].

Moreover, fermented foods appear to be beneficial against viruses. Natto produced with *Bacillus subtilis* can inhibit virus reproduction, including SARS-CoV-2. In an in vitro study on bovine BHV-1 cells, this fermented soy product inhibited viral infection through the proteolysis of virus proteins. This was probably due to the natto’s serine-protease(s) activity [125].

As of today’s knowledge, fermented vegetables don’t exhibit an adverse influence on the human immune system. Furthermore, by influencing gene expression levels, these foods appear to be beneficial and anti-inflammatory.

### 8.3. Antioxidant Function

Fermented vegetables, due to their high content of bioactive compounds, vitamins, and mineral components, can support the treatment of many diseases. Polyphenols, found in high amounts in these foods, reduce oxidative stress biomarkers, cancer, and cardiovascular disease risk [126].

Pickled products, such as kimchi, modulate the immune response by regulating the secretion of interleukin-6 (IL-6), which is associated with cachexia in cancer. It was found that consumption of kimchi significantly attenuated the development of cancer cachexia, as manifested by less loss of body weight and muscle tissue, as well as higher survival rates for mice. This was associated with the inhibition of IL-6 and its signaling. Administration of kimchi provided significant inhibition of the muscle-specific ubiquitin-proteasome system, which prevented cell death [127]. In addition, antioxidants from kimchi were characterized by preventive effects against neurodegenerative diseases [128]. Due to its potential to reduce oxidative stress and scavenge free radicals, kimchi consumption has been linked to slowing the aging process. This traditional Asian dish caused a reduction in hydrogen peroxide-induced oxidative stress and inhibition of lipid peroxidation [129].

In addition, pickled vegetables may improve depression and anxiety disorders by reducing intestinal permeability, attenuating inflammation, and affecting the brain–gut axis and the hypothalamic–pituitary–adrenal axis. However, this topic requires more clinical studies to definitely confirm the importance of fermented products [130,131].

Most common disorders in civilized countries, such as cancer or depression, appear to be caused by stress, reactive oxygen species (ROS), and a disrupted immune system [132,133]. A decrease in oxidative stress via consumption of fermented products is a simple way to prevent many lifestyle diseases. However, there is still a lack of research enabling a clear confirmation of this hypothesis.

### 8.4. Metabolic Function

Fermentation is an effective way to produce and increase the amount of bioactive peptides in food products [70]. Lactic acid bacteria, such as *Lactococcus lactis*, *Lactobacillus helveticus* and *Lactobacillus delbrueckii* ssp. *Bulgaricus*, are mainly responsible for the production of peptides with ACE inhibitory activity. These compounds may contribute to lower blood pressure in a group of spontaneously hypertensive animals [132].

Fermented foods may also be relevant to other metabolic diseases. Consumption of kimchi can be associated with improved glucose-insulin metabolism and anthropometric parameters in a group of individuals with an increased risk of developing type 2 diabetes [133,134]. Consumption of tempeh with LAB results in alterations in the internal microbiota, leading to the inhibition of cholesterol synthesis and promotion of lipolysis in rats [135]. Fermented foods have also been associated with effects on body fat in people. A double-blind randomized study showed that consumption of chungkookjang, a dish made from fermented soybeans, can reduce body fat percentage, increase lean body mass, and improve the waist-to-hip circumference ratio in a group of women. Additionally, improvements in lipid profile and atherosclerotic indices were observed in the same group [136]. Fermented soy products were also characterized by fibrinolytic properties [137]. Another benefit associated with fermented products’ consumption is the prevention of osteoporosis, especially in postmenopausal women. Such properties were attributed to natto, mainly due to its high menaquinone-7 content [138].

Additionally, fermentation allows people with gastrointestinal problems to consume foods that cause bloating. The ability of microorganisms to synthesize the enzyme β-glucosidase allows the hydrolysis of soybean oligosaccharides, thereby reducing the risk of flatulence commonly occurring after the consumption of legumes and their products [139]. That feature may be used to enrich the menu of people on an elimination diet.

The metabolic function of fermented vegetables is connected with their antioxidant function. The ability to reduce oxidative stress decreases the risk of lifestyle diseases, including obesity, atherosclerosis, and hypertension. This is a very important feature of fermented vegetables. Epidemics of these disorders were observed in the 21st century. It turns out that a few changes in a daily diet and the introduction of silages to the menu can prevent many dangerous diseases and, thus, extend life.

### 8.5. Health Risk Related to High Salinity of Fermented Vegetables

Salt (NaCl) or salt substitutes are always present in fermented vegetables [3,4,9,140]. Every flavoring substance (NaCl, KCl, monosodium glutamate) has a different set of health risks.

The health impact of sodium chloride is widely recognized due to its role in increasing hypertension [141,142]. According to the Food and Agriculture Organization and the World Health Organization (FAO/WHO), overall consumption of NaCl should not exceed 5 g/day [143]. The greatest consumption of sodium chloride comes from silages and is probably noted in Korea. That is due to the high intake of kimchi. According to a population study, the average Korean consumes over 5 g of NaCl/day, of which 19.6% comes from kimchi [144]. Nevertheless, consumption of fermented vegetables (kimchi) was not associated with this disease prevalence [145,146]. Moreover, these food products can exhibit an anti-hypertensive effect due to active biological substances such as ACE inhibitors. Such a link was demonstrated in mice [147]. Due to the probable, but not confirmed, adverse effect of fermented foods on hypertension, potassium salts are used as sodium salt substitutes for the process in question [148]. Potassium chloride is a long-known substitute for NaCl in the fermentation process and is used in various proportions [4]. Partial replacement of NaCl by KCl in fermented foods was associated with a lower blood pressure of about 5.58 mm Hg in people. Moreover, 30% or less of KCl was considered the best proportion in this aspect [149]. However, the greatest concern with potassium-enriched salt substitutes is the potential for adverse influence due to hyperkalemia [150]. Until today, such a link between hyperkalemia and consumption of fermented vegetables produced with KCl in people with fine kidney function has not been confirmed [149]. There is a lack of research on patients with kidney failure. However, people with diagnosed kidney failure have to limit their potassium intake in the diet. As a result, people with this disorder should limit their intake of silages [151].

**Figure 3:**
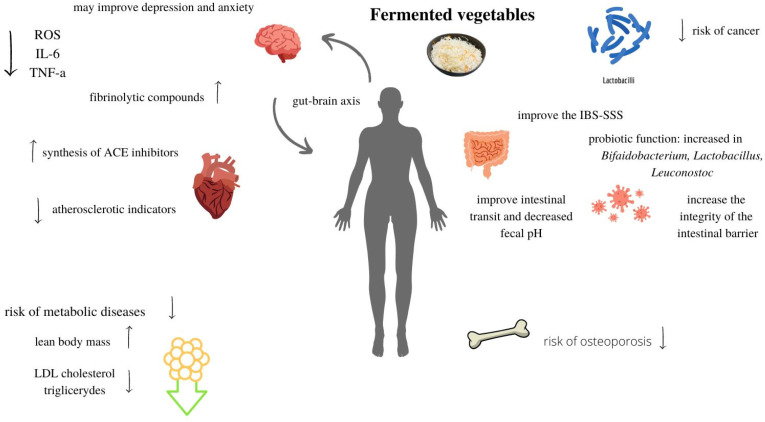
Summary of health effects of fermented vegetables. ROS—reactive oxygen species, IL-6—interleukin 6, TNF-α—tumor necrosis factor-α. The up and down arrows indicate the directions of changes in the body after consuming fermented vegetables.

There is little evidence of NaCl and KCl consumption with fermented vegetables. Until today, hypertension prevalence has not been connected with silage consumption [144,145]. Moreover, fermented vegetables produced with KCl as a salt substitute can even decrease blood pressure. However, due to the potential negative health effects of this substance, more data on this topic is required. There is no data on how increased consumption of silages based on NaCl affects patients already diagnosed with hypertension. Fermented vegetables exhibit pleiotropic effects on the body (Figure 3). Such foods are not only relevant to the microbiome and its combined effects but can also ameliorate adverse effects associated with civilizational problems affecting health. These include reducing inflammation, the risk of cancer, or metabolic diseases such as type 2 diabetes, as well as slowing aging [119]. However, there are possibilities of their adverse influence on human health in cases of hypertension or kidney failure. There is a need for deeper research on this topic, especially considering the higher interest in these products among consumers.

## 9. Conclusions

Fresh vegetables should be the basis of a healthy and balanced diet. However, fermented products are characterized by even higher health potential. Changes that occur during microbiological transformation are determined by the specificity of the raw material, as well as the starter culture used in controlled fermentation. Specific conditions of this process may affect the final product. For example, 1.5% of NaCl is the best concentration to decrease the loss of vitamin C. Moreover, culinary treatment such as heating over 65 °C (e.g., pasteurization) significantly reduces the viability of microorganisms and probiotic strains in silages.

There are plenty of changes in food products during fermentation. Most of them seem to be beneficial. Specific changes depend on the fresh sample, but there are several features that are common for the majority of fermented vegetables. The most important one, affecting the bioavailability of nutrients from these foods, is the decrease in anti-nutritional compounds, such as tannins or phytates. That is due to an enzyme produced by bacteria (β-glucosidase) which releases micronutrients and antioxidants from conjugated bonds with them. This is possible due to the activity of some bacterial species, such as *Lactobacillus acidophilus*, *Bifidobacterium lactis*, or *Lacticaseibacillus casei*. This results in an increased daily intake of elements such as Ca, Fe, and Zn and bioactive components such as phenols and isoflavones. Reduced quantities of anti-nutrients may also influence macronutrients—specifically, protein. This component in fermented legumes is characterized by improved digestibility and a higher amount of essential amino acids. This is an important feature, especially for people on vegan diets exposed to protein deficiencies. Moreover, fermented vegetables are characterized by higher amounts of vitamin K, riboflavin, thiamine, cobalamin, and folate. These substances are synthesized during both controlled and spontaneous fermentation. In the first type, *Bacillus subtilis* natto was identified as the greatest producer of riboflavin and vitamin K. The best producers of vitamins during spontaneous fermentation have not been determined yet. Other bioactive substances are also present in fermented vegetables in higher amounts than in fresh samples. This is primarily due to specific microorganisms that form during the fermentation process acting as their producers. Most of them were linked to specific strains and environmental conditions. *Lactobacillus buchneri* and other *Lactobacillus* bacteria were identified as the greatest producers of GABA. Furthermore, the highest productivity was observed when the pH was slightly acidic, 4.2, and the NaCl concentration was 2%. Additionally, fermentation can increase the microbiological safety of products. This is due to the presence of LAB producing lactic acid and lowering the pH of the final product so that pathogenic microorganisms are unable to survive.

However, despite the formation of new bioactive compounds during the fermentation of vegetables, there are still no data about their fate in the gastrointestinal tract. Future research should focus on how, in what form, and with what bioavailability these substances are characterized after the consumption of fermented vegetables. Innovative products are constantly appearing in the food market, including pickled vegetables that have not been studied enough. Due to the importance of the characteristics of the fresh sample on the final composition of the fermented product, this group of foods should be carefully analyzed.

Nevertheless, there are also adverse effects of fermentation. They mainly concern vitamin C and fiber. A decrease in vitamin C is one of the most frequent changes during this process. However, according to existing research, it does not have any adverse impact on general antioxidant potential. Fiber is used by microorganisms as an energy source. Therefore, a lower content of this component is observed in fermented vegetables. However, there is a decrease in starch and an increase in resistant starch, resulting in a lower GI. That is a beneficial change, especially for people with glycemic disorders.

If it concerns health effects, fermented vegetables are considered to be beneficial. Despite the low viability of microorganisms in the gastrointestinal tract, the probiotic potential of products such as kimchi and sauerkraut was repeatedly confirmed. Lactic acid bacteria were found in increased amounts in feces after consumption of these food. Furthermore, when discussing the rising number of diseases characteristic of civilized countries, such as cancer or depression, antioxidant function via regulation of interleukin secretions and its impact on expression gene levels is critical. However, the high salt content in silages may be questionable, but to the best of our knowledge, consumption of fermented products is not connected with hypertension. On the contrary, several studies show that these foods can be associated with a lower risk of many lifestyle diseases, including high blood pressure.

A low number of studies on fermented vegetables prevents the creation of solid statistical data that could clearly determine the best conditions for vegetables’ fermentation in order to obtain a product of the highest health quality. Without a doubt, there should be research performed concerning all pickled vegetables, which are globally available. These studies should focus on every aspect, including the content of various ingredients, their bioavailability from products, and their influence on human health and gut microbiota.

## Figures and Tables

**Table 1:** Macronutrients’ content in the selected fresh and fermented vegetables [g/100 g] based on [19].

Vegetables	Proteins	Carbohydrates	Fibre	Total Fats	SFA	MUFA	PUFA
Beetroot	1.61	9.56	2.8	0.17	0.027	0.032	0.06
Beetroot, fermented	0.8	16.3	0.8	0.08	0.013	0.016	0.029
Chickpea, canned, no added fat	8.19	22.4	7.3	3.24	0.339	0.739	1.47
Soybean, cooked	16.9	7.77	5.6	14.8	2.1	4.57	7.39
Natto	19.4	12.7	5.4	11	1.59	2.43	6.21
Red cabbage	1.43	7.37	2.1	0.16	0.021	0.012	0.08
Red cabbage, pickled	1.19	11	1.7	0.15	0.017	0.01	0.066
Lentils, cooked	8.38	18.7	7.3	6.86	0.939	2.78	2.84
Fava beans, cooked	7.06	18.3	5	6.88	0.951	2.8	2.83

SFA—saturated fatty acids, MUFA—monounsaturated fatty acids, PUFA—polyunsaturated fatty acids.

**Table 2:** Mineral components, vitamin C, riboflavin, and folates contents in the selected fresh and fermented vegetables, based on [19].

Vegetables	Ca [mg/100 g]	Fe [mg/100 g]	Mg [mg/100 g]	Zn [mg/100 g]	Vitamin C [mg/100 g]	Riboflavin [mg/100 g]	Folates, Total [µg/100 g]
Beetroot	16	0.8	23	0.35	4.0	0.04	109
Beetroot, pickled	11	0,41	15	0.26	2.3	0.048	27
Green cabbage	40	0.47	12	0.18	36.6	0.04	43
Sauerkraut, Japanese style	35	0.39	10	0.15	25.8	0.032	30
Radish	25	0.34	10	0.28	14.8	0.039	25
Radish, pickled, Hawaiian style	22	0.29	8	0.23	10.4	0.032	18
Carrot	33	0.3	12	0.24	5.9	0.058	19
Cucumber	16	0.28	13	0.2	8	0.033	7
Cucumber, pickled	54	0.23	7.1	0.11	2.1	0.057	8
Parsley, root	138	6.2	27	1.07	45	0.086	180
Red cabbage	45	0.8	16	0.22	57	0.069	18
Red cabbage, pickled	39	0.67	13	0.18	40.2	0.055	13
Green tomato, raw	13	0.51	10	0.07	23.4	0.04	9
Green tomato, pickled	12	0.43	8	0.06	18.4	0.032	5

## Data Availability

All data is contained within the article.
